# Organ-on-Chip Approaches for Intestinal 3D In Vitro Modeling

**DOI:** 10.1016/j.jcmgh.2021.08.015

**Published:** 2021-08-25

**Authors:** Joana Pimenta, Ricardo Ribeiro, Raquel Almeida, Pedro F. Costa, Marta A. da Silva, Bruno Pereira

**Affiliations:** 1 i3S - Instituto de Investigação e Inovação em Saúde, Universidade do Porto, Porto, Portugal; 2 IPATIMUP - Instituto de Patologia e Imunologia Molecular da Universidade do Porto, Porto, Portugal; 3 BIOFABICS, Porto, Portugal; 4 FMUP - Faculdade de Medicina da Universidade do Porto, Porto, Portugal; 5 Departamento de Biologia, FCUP - Faculdade de Ciências da Universidade do Porto, Porto, Portugal; 6 Present address: IBMC - Instituto de Biologia Molecular e Celular, Universidade do Porto, Porto, Portugal

**Keywords:** Intestinal Stem Cells, Organoids, Microfabrication, Intestine-on-Chip, 2D, 2-dimensional, 3D, 3-dimensional, BMP, bone morphogenetic protein, ECM, extracellular matrix, ENS, enteric nervous system, HIF, hypoxia-inducible factor, IBD, inflammatory bowel disease, iPSC, induced pluripotent stem cell, ISC, intestinal stem cell, LGR5, leucine-rich repeat-containing G-protein–coupled receptor 5, PBMC, peripheral blood mononuclear cells, PEG, polyethylene glycol, YAP, yes-associated protein

## Abstract

The intestinal epithelium has one of the highest turnover rates in the human body, which is supported by intestinal stem cells. Culture models of intestinal physiology have been evolving to incorporate different tissue and microenvironmental elements. However, these models also display gaps that limit their similarity with native conditions. Microfluidics technology arose from the application of microfabrication techniques to fluid manipulation. Recently, microfluidic approaches have been coupled with cell culture, creating self-contained and modular in vitro models with easily controllable features named organs-on-chip. Intestine-on-chip models have enabled the recreation of the proliferative and differentiated compartments of the intestinal epithelium, the long-term maintenance of commensals, and the intraluminal perfusion of organoids. In addition, studies based on human primary intestinal cells have shown that these systems have a closer transcriptomic profile and functionality to the intestine in vivo, when compared with other in vitro models. The design flexibility inherent to microfluidic technology allows the simultaneous combination of components such as shear stress, peristalsis-like strain, 3-dimensional structure, oxygen gradient, and co-cultures with other important cell types involved in gut physiology. The versatility and complexity of the intestine-on-chip grants it the potential for applications in disease modeling, host-microbiota studies, stem cell biology, and, ultimately, the translation to the pharmaceutical industry and the clinic as a reliable high-throughput platform for drug testing and personalized medicine, respectively. This review focuses on the physiological importance of several components that have been incorporated into intestine-on-chip models and highlights interesting features developed in other types of in vitro models that might contribute to the refinement of these systems.

**Summary**

This review focuses on the different biological parameters that advanced 3-dimensional intestinal in vitro models, particularly intestine-on-chip systems, should incorporate to better mimic the full complexity of the intestinal tissue and microenvironment.

The intestine is a critical organ in the digestive system, mainly because of its role in nutrient absorption. Intestinal microarchitecture (including plicae circulares, villi, and microvilli) is designed specifically to increase surface area and therefore enhance the efficiency of the absorptive processes.^1^ However, the intestinal luminal microenvironment is quite hostile, with constant exposure to low levels of pH, microorganisms, and xenobiotics. Therefore, the intestinal epithelium has one of the highest turnover rates in the human body,^2^ also exhibiting a fast and reliable mechanism of regeneration upon mucosal injury. This is ensured by a population of resident intestinal stem cells (ISCs) located within submucosal invaginations called crypts of Lieberkühn. At the bottom of the crypts, ISCs are shielded from the aggressive luminal environment and protected from pathogenic agents by surrounding Paneth cells that secrete antimicrobial peptides.

Over the past years, different ISC markers have been described, with the leucine-rich repeat-containing G-protein–coupled receptor 5 (LGR5) being the most well known. LGR5+ cells are an intestinal multipotent stem cell population, showing self-renewing activity and the potential for originating absorptive, goblet, Paneth, enteroendocrine, microfold, and tuft cells.^3^^,^^4^ LGR5+ cells are found primarily at the bottom of the crypt, interspersed with Paneth cells. Each ISC divides every 24 hours and can give rise to transit-amplifying cells and/or other ISCs. Although the latter remain undifferentiated at the bottom of the crypt, the former move upward on the epithelial layer in direction of the villus top, while becoming differentiated.^5^ The ISC niche gathers several signaling molecules and growth factors necessary to maintain the stem cell population and ensure continuous proliferation and regeneration of the epithelium.^4^ In fact, signaling pathways such as Wnt, Notch, and bone morphogenetic protein (BMP) form opposing morphogenic gradients along the crypt/villus axis that are responsible for creating compartmentalized proliferative (crypt) and differentiated (villus) zones. Paneth and subepithelial mesenchymal cells are among the main contributors to the maintenance of the ISC niche.

The understanding of the intestinal function and homeostatic process is fundamental for modeling disease conditions and the development of novel therapeutic approaches. In this context, several in vitro and in vivo models are being used in the laboratory to mimic intestinal features as closely as possible. Recently, gut organoids have emerged as a promising platform for biomedical research and applications.^6^ Although organoids usually are referred to as mini-organs, their simplistic structure does not fully mimic a complex organ. Therefore, biofabrication techniques have been used to develop devices that can surpass organoid limitations. In this regard, microfluidic devices recently emerged as interesting platforms for in vitro models of human organs. The perfusion created in microfluidic devices mimics the function of blood circulation in tissues, allowing communication between different tissues and biochemical environments through microchannels or porous membranes.^7^ The features and structure of microfluidic devices are completely tunable so that a variety of components of a system can be included for a better representation of complex physiological environments. Taking this range of possibilities into account, microfluidic devices permit a reductionist representation of an organ’s functions, creating organ-on-chip systems. Organs-on-chip are designed considering the characteristics of the organ’s functional units; therefore, they include the different cell types of the organ, recapitulate the structural organization, as well as the organ-specific physical and biochemical microenvironment. As a result, organ-on-chip platforms provide a more precise control of cell culture.^8^ Since the first intestine-on-chip was developed in 2008 by Kimura et al,^9^ a number of intestines-on-chip have been reported in the literature (Table 1). These models, designed to mimic intestinal structure and physiology, have been providing valuable insights regarding host-microbiota interaction,^10^, ^11^, ^12^ intestinal inflammatory diseases,^13^, ^14^, ^15^ and are becoming relevant platforms for pharmacologic assays^16^, ^17^, ^18^ and organoid cultures.^19^^,^^20^ The main advantage of intestine-on-chip models is the incorporation of dynamic fluid flow, which can be applied to simulate blood circulation, basally supplying epithelial cells with nutrients and growth factors as well as intraluminal flow, mimicking the circulation of nutrients, drugs, and pathogens inside the intestine.

**Table 1:** Main Features and Components of Intestine-on-Chip Models Reported in the Literature

Reference	Intestinal cells	Co-culture	Peristalsis simulation	Oxygen gradient	Device material	Membrane material and ECM	Fabrication technique	Major observations	Main parameter tested
Kimura et al, 2008^9^	Caco-2	No	No	No	PDMS	Polyester, type I collagen	Photolithography	Allowed long-term culture (up to 30 days), induced polarized transport activity	Microfluidics and mechanical stimulation
Kim et al, 2012^10^	Caco-2	*L rhamnosus*	Cyclic strain	No	PDMS	PDMS, type I collagen and Matrigel mix	Soft lithography	Fluid flow accelerated intestinal epithelial differentiation and organization into villi-like structures, mechanical stimulation enhanced specific differentiation features, sustained long-term co-culture with commensal bacteria	
Kim and Ingber, 2013^21^	Caco-2	No	Cyclic strain	No	PDMS	PDMS, type I collagen and Matrigel mix	Soft lithography	Observed increased proliferative activity at the base of villi-like structures and differentiation into the 4 major intestinal epithelial cell types	
Pocock et al, 2017^18^	Caco-2	No	No	No	PDMS	Polycarbonate, Matrigel	Soft lithography	Defined a permeability coefficient across the intestinal barrier for lipophilic drugs	
Triestch et al, 2017^22^	Caco-2	No	No	No	Glass, polystyrene, and proprietary polymers	Membrane-free (PhaseGuide; MIMETAS,Leiden, the Netherlands), type I collagen	Not described	Established a high-throughput platform with pumpless flow, showed its suitability for epithelial barrier integrity studies, first commercially available model	
Guo et al, 2018^23^	Caco-2	No	No	No	PDMS	Nitrocellulose, type I collagen	Soft lithography	Engineered a 4-parallel cell culture chamber device, evaluated drug metabolism (exposure to verapamil and ifosfamide)	
Workman et al, 2018^24^	iPSC-derived human intestinal organoids and Caco-2	No	No	No	PDMS	PDMS, Matrigel	Soft lithography	Showed the feasibility of intestinal organoid–derived culture on a microengineered chip, continuous luminal flow led to the development of villus-like projections, the culture system was biologically responsive to inflammatory cytokines	
Sidar et al, 2019^19^	iPSC-derived human intestinal organoids	No	No	No	Polymethyl methacrylate	Membrane-free (central well), Matrigel	Laser cutting	Established luminal and extraluminal flow in single intestinal organoids, cell viability was unaffected by long-term porting and luminal flow	
Kasendra et al, 2018^25^	Human duodenal organoids (pediatric donors)	HIMECs	Cyclic strain	No	PDMS	PDMS, type I collagen and Matrigel mix	Soft lithography	Transcriptome of the intestinal tissue-on-chip more closely resembled that of the duodenum in vivo than the initial organoid culture from which it was derived, co-culture with endothelial cells accelerated the formation of the epithelial monolayer	
Kasendra et al, 2020^16^	Human duodenal organoids (adult donors)	HIMECs	Cyclic strain	No	PDMS	PDMS, type IV collagen and Matrigel mix (epithelial side), type IV collagen and fibronectin mix (vascular side)	Soft lithography	Showed culture system suitability for studying intestinal metabolism and drug transport	
Yin et al, 2020^26^	Human jejunal organoids (adult donors)	HUVECs	Cyclic strain	No	PDMS	PDMS, type IV collagen	Soft lithography	Shear stress generated by luminal and basolateral flow produced a model of continuous intestinal differentiation, no villi-like structures observed with stem cell expansion media on the luminal side	
Shin et al, 2019^27^	Human colon organoids (adult donors) and Caco-2	No	No	No	PDMS	PDMS, type I collagen and Matrigel mix	Soft lithography	Observed that fluid flow was more determinant than mechanical deformation for induction of 3D morphogenesis, removal of basolaterally secreted Wnt antagonists, such as DKK1, rapidly triggered villi-like intestinal morphogenesis mediated by FZD9	
Sontheimer-Phelps et al, 2020^28^	Human colon organoids (pediatric and adult donors)	No	No	No	PDMS	PDMS, type I collagen and Matrigel mix	Soft lithography	Replicated the colonic mucus bilayer, spontaneous differentiation of mucus-producing MUC2+ goblet cells observed at similar levels to those present in the human colon in vivo	
Richardson et al, 2020^29^	Mouse colon tissue explant	Intestinal submucosal and muscular layers, microbiota	No	Yes	Cyclin olefin polymer and polyurethane	Membrane-free, no ECM	Injection molding	Dual-flow microfluidics allowed for the culture of full-thickness explants over 3 days, recapitulated the in vivo oxygen gradient across the epithelial layer	
Shim et al, 2017^17^	Caco-2	No	No	No	PDMS, PET, and glass	Collagen 3D scaffold	Soft lithography and micromolding	Combined a 3D collagen scaffold mimicking human intestinal villi with fluidics, the culture system enhanced metabolic activity	Architectural cues
Costello et al, 2017^30^	Caco-2	No	No	No	VeroClear-RGD810 (Stratasys, Minneapolis, MN)	Polyethylene-vinyl-acetate 3D scaffold	3D printing and micromolding	Bioreactor culture with a villi polymeric scaffold led to cell differentiation and apoptosis gradients, observed physiological levels of glucose absorption	
Nikolaev et al, 2020^20^	Mouse proximal small intestine organoids	*Cryptosporidium parvum*	No	No	PDMS	Type I collagen and Matrigel mix–coated 3D scaffold	Soft lithography and laser ablation	Established a long-lived and tube-shaped intestinal epithelial culture system by using crypt-like microcavities under flow, induced topography-guided self-organization of a functional epithelium with crypt- and villus-like domains similar to that observed in vivo, the culture system showed self-regeneration capacity and response to bacterial infection	
Shah et al, 2016^12^	Caco-2	*L rhamnosus* GG and *Bacteroides caccae*	No	Yes	Polycarbonate	Polycarbonate, type I collagen (epithelial chamber) and porcine gastric mucin (microbial chamber)	Computer-controlled milling, laser cutting, and bolting	Engineered a modular architecture consisting of 3 microchambers to facilitate human and microbial cell interface, allowed measuring individual transcriptional responses in different infectious contexts and real-time monitoring of oxygen concentrations	Microbiota co-culture
Kim et al, 2016^13^	Caco-2	Human gut microbiota, *E coli*, human PBMCs, human microvascular endothelial cells, and human lymphatic microvascular endothelial cells	Cyclic strain	No	PDMS	PDMS, type I collagen and Matrigel mix	Soft lithography	Established a stable long-term co-culture system of commensal and pathogenic microbes with intestinal epithelial cells, lack of mechanical stimulation induced bacterial overgrowth, similar to what is observed in IBD patients, emulated intestinal infection and inflammatory responses	
Shin and Kim, 2018^14^	Caco-2	Human gut microbiota, *E coli*, PBMCs	Cyclic strain	No	PDMS	PDMS, type I collagen and Matrigel mix	Soft lithography	Re-created a dextran sodium sulfate–induced epithelial inflammatory response, described intestinal barrier dysfunction as a critical trigger of inflammation onset in the gut	
Grassart et al, 2019^31^	Caco-2	*S flexneri*	Cyclic strain	No	PDMS	PDMS, type I collagen and Matrigel mix	Soft lithography	Enabled the replication of *Shigella* infection hallmarks, *Shigella* invaded directly via the luminal side of the epithelium composed solely of enterocytes, 3D crypt-like structures provided a safe harbor for bacteria against luminal washout	
Tovaglieri et al, 2019^32^	Human colon organoids (pediatric and adult donors)	HIMECs, EHEC	No	No	PDMS	PDMS, type I collagen and Matrigel mix	Soft lithography	Observed that infectious activity of EHEC is promoted by human gut microbiome metabolites, when compared with those derived from mouse, recapitulated the proinflammatory and anti-inflammatory cytokine profiles induced by EHEC infection	
Sunuwar et al, 2020^33^	Human jejunal organoids (adult donors)	No	Cyclic strain	No	PDMS	PDMS, type IV collagen	Soft lithography	Flow and mechanical strain increased extracellular cyclic guanosine monophosphate secretion in response to EHEC-produced heat-stable enterotoxin A	
Shin et al, 2019^34^	Caco-2	*Bifidobacterium adolescentis* and *Eubacterium hallii*	Cyclic strain	Yes	PDMS	PDMS, type I collagen and Matrigel mix	Soft lithography	Simulated a steady-state vertical oxygen gradient, the transepithelial anoxic–oxic interface allowed co-culture with obligate anaerobes	Oxygen gradient
Jalili-Firoozinezhad et al, 2019^11^	Caco-2 and human ileal organoids (pediatric donors)	*Bacteroides fragilis*, human gut microbiota, HIMECs	Cyclic strain	Yes	PDMS	PDMS, type I collagen and Matrigel mix	Soft lithography	Established an oxygen gradient compatible with co-culture of a complex community of anaerobic commensal microorganisms	
Beaurivage et al, 2019^35^	Caco-2	No	No	No	Glass, polystyrene, and proprietary polymers	Membrane-free (PhaseGuide), type I collagen	Refer to Triestch et al, 2017^22^	Simulated immune activation of Caco-2 cells to mimic physiological aspects of IBD pathology, monitored barrier integrity in real time	Immune response
Maurer et al, 2019^15^	Caco-2	HUVECs, PBMCs, mucosal macrophages, dendritic cells, *L rhamnosus*, *Candida albicans*	No	No	Polystyrol	PET	Injection molding	Characterized immunologic responses to luminal lipopolysaccharide and endotoxemia, addressed the role of probiotics in protecting from opportunistic infections	
Gjorevski et al, 2020^36^	Caco-2	Neutrophils, monocytic THP1 cells	No	No	Glass, polystyrene, and proprietary polymers	Membrane-free (PhaseGuide), type I collagen	Refer to Triestch et al, 2017^22^	Simulated acute intestinal inflammatory responses by enabling neutrophil recruitment to the parenchymal compartment	
Beaurivage et al, 2020^37^	Human colon organoids (pediatric and adult donors)	PBMCs	No	No	Glass, polystyrene, and proprietary polymers	Membrane-free (PhaseGuide), type I collagen	Refer to Triestch et al, 2017^22^	Studied IBD-associated inflammatory responses	
Seiler et al, 2020^38^	Human ileal organoids (pediatric donors)	Endothelial colony-forming cell-derived endothelial cells, intestinal subepithelial myofibroblasts	No	No	PDMS	Polycarbonate, type IV collagen	Soft lithography	Characterized ISEMF-induced angiogenesis and its physiological parameters, evaluated the effect of perfused vasculature on intestinal epithelial cell culture	Vasculature

DKK1, dickkopf Wnt signaling pathway inhibitor 1; ECM, extracellular matrix; EHEC, enterohemorrhagic *E coli*; FZD9, frizzled class receptor 9; HIMEC, human intestinal microvascular endothelial cell; HUVEC, human umbilical vein endothelial cell; ISEMF, intestinal subepithelial myofibroblast; MUC2, mucin 2; PDMS, polydimethylsiloxane; PET, polyethylene terephthalate; THP1, Tohoku Hospital Pediatrics-1 cell line.

This review focuses on the physiological importance of various biological features and components that increasingly are being incorporated into intestine-on-chip devices and suggests other relevant elements that should be considered when constructing advanced 3-dimensional (3D) cell culture models of the human intestine (Figure 1).

**Figure 1:**
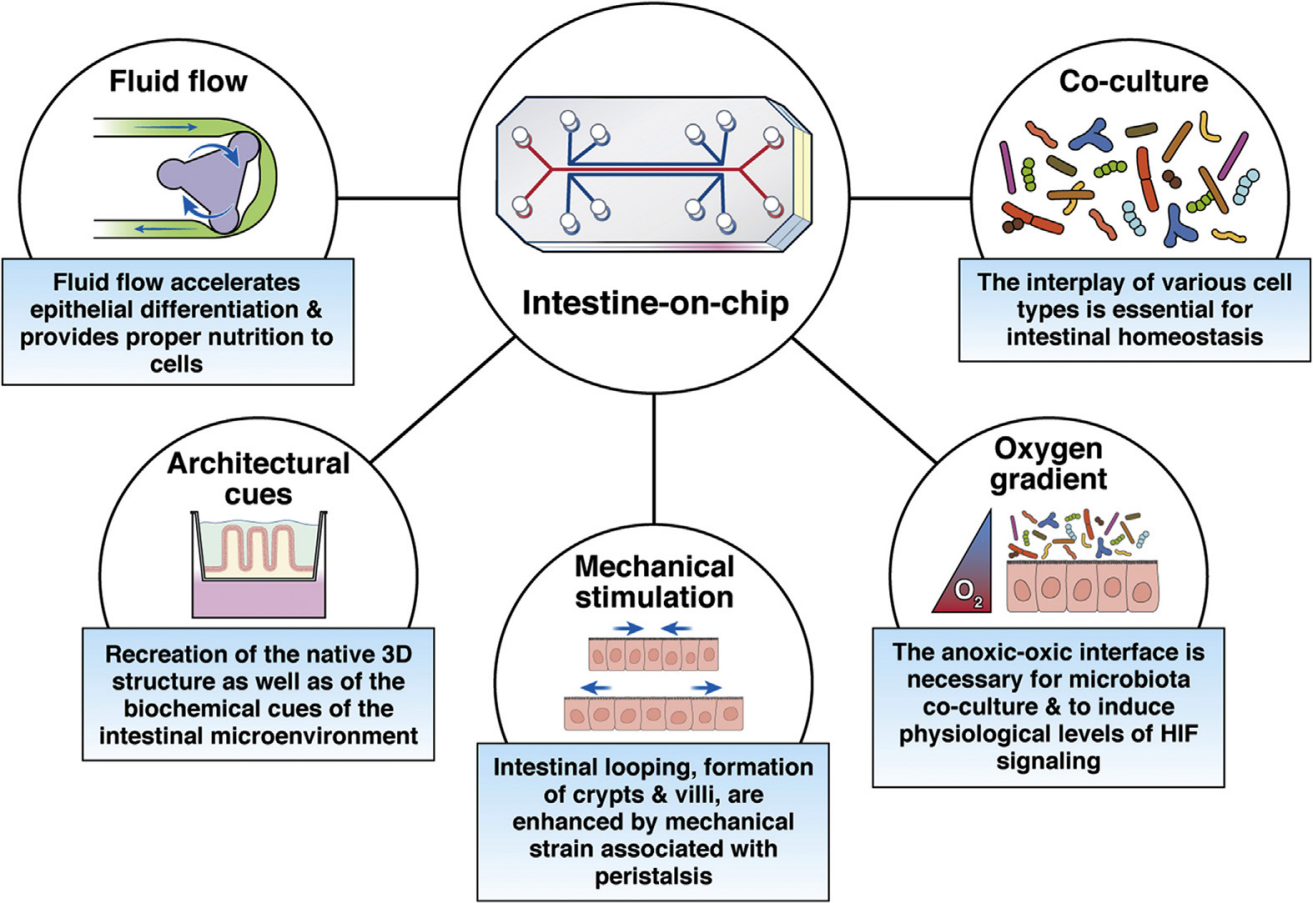
**Different features of intestine-on-chip models.** These individual elements can be incorporated into single intestine-on-chip models to increase their complexity and provide a more faithful reconstruction of the intestinal tissue and microenvironment. 3D, 3-dimensional; HIF, hypoxia-inducible factor.

## Conventional Intestinal In Vitro Models

The interest in in vitro models of the intestine is widely spread across different fields, with applications in pharmacology, nutrition, microbiology, immunology, pathology, and developmental studies. Culture of intestinal epithelial cells in Transwell insert systems has been the gold standard for a variety of studies in this context, being commonly used for permeability and pharmacologic assays during drug preclinical testing.^39^ These systems commonly display a static nature, without media flow or applied mechanical strain. Formation of both crypt- and villi-like structures is not attainable, indicating that epithelial morphogenesis is not fully recapitulated, thus raising questions about the reliability of the results obtained. Another important issue with most of these models is the use of the heavily modified Caco-2 cells, an immortalized cell line derived from human colorectal adenocarcinoma.^40^ Although experiments using established cell lines have various advantages such as cost effectiveness, unlimited supply, and longer periods of culture, these cells are derived from tumors and present genetic and phenotypic abnormalities that significantly limit their physiological relevance.

The development of 3D models of the intestine is particularly important because of the unique architectural layout of this tissue and its impact on functionality. A major leap in the field was the discovery of the minimal growth factor requirements to generate and maintain multicellular structures known as intestinal organoids from isolated crypts and single LGR5+ cells. These organoids display conspicuous crypt domains surrounding a central lumen, lined by a villus-like epithelium. Soon, mini-gut cultures started to be established from different sources of human tissue. The directed differentiation of embryonic stem cells and induced pluripotent stem cells (iPSCs) into intestinal organoids is an appealing strategy, providing unlimited cell supply and the added capacity to generate both epithelial and stromal cell populations.^41^^,^^42^ However, these methods are time-consuming and entail convoluted experimental conditions that often lead to the development of intestinal tissue with immature (fetal) characteristics.^43^ On the other hand, the use of adult tissue as a source of multipotent stem cells already committed to the intestinal identity is the most practical approach. Regardless, intestinal organoids can be cultured long term (at least >1 year) by weekly passages while maintaining a stable karyotype and phenotype, can be cryopreserved, and can be engrafted in vivo. Such properties highlight their vast potential as platforms for disease modeling, drug testing, and, possibly, regenerative medicine. However, several limitations still exist in classic intestinal organoid culture settings such as the presence of only the epithelial component, access to nutrients and oxygen strictly by diffusion, presence of an enclosed lumen that renders access to the apical surface experimentally challenging, dependence on a cocktail of growth factors provided in media, and their heterogenous shape and size.

To better re-create the intestinal microenvironment, several research groups have developed more complex in vitro models, based on organ-on-chip technologies, in which different culture parameters are being integrated.

## Microfluidics

In conventional cultures, cells are maintained under static conditions in which dynamic parameters cannot be mimicked easily. In microfluidic devices, culture medium can be perfused at defined flow rates, replicating the in vivo ranges of fluid flows and their associated shear stresses over the cell surface. This allows increased control over the culture microenvironment, including the establishment of physiological gradients of nutrients and growth factors. In these platforms, fluid flow can be generated by peristaltic pumps,^12^^,^^15^ syringe pumps,^10^^,^^21^ gravity or hydrostatic pressure,^22^^,^^38^ and pressure generators.^44^ These methods vary in terms of their ease of operation, flow rate precision control, and the level of pressure fluctuation during operation.

A pumpless system based on plate tilting was reported to re-create intestinal intraluminal and extraluminal flow, although not in constant perfusion. Still, studies have shown that this system induced proper polarization of Caco-2 cells, evidenced by the presence of tight junctions and brush borders.^22^ Although these systems have an easy, high-throughput setup, these models are limited in terms of re-creating defined flow rates. Based on previous designs, Kim et al^10^ reported in 2012 an intestine-on-chip model with fluid flow and applied cyclic mechanical strain. The device was composed of 2 microchannels sided by 2 hollow chambers where vacuum was applied, causing the porous membrane separating the channels to elongate unidirectionally. In this seminal work, the combination of fluid flow and cyclic mechanical strain resulted in the formation of folds that recapitulate the structure of intestinal villi. Surprisingly, the Caco-2 cells used in the system displayed a polarized columnar morphology and were similar in size to epithelial cells in vivo (30–40 μm high). The 5-ethynyl-2′-deoxyuridine pulse-chase experiments showed that proliferative activity was restricted to the basal regions between adjacent villi and labeled cells migrated upward, suggesting that undifferentiated cells remain in the crypt-like niche and generate daughter cells that become more differentiated along the villus axis.^21^ This design also allows primary evaluation of drug metabolism in the intestinal setting.^23^ Moreover, the device is compatible with the culture of intestinal epithelial cells and organoid fragments derived from primary sources,^11^^,^^16^^,^^19^^,^^24^, ^25^, ^27^ which show a stronger resemblance in transcriptomic profile to the intestine in vivo.^25^ In addition, these in vitro models display similar percentages of the different epithelial cell types to the native organ and support the formation of a dynamic mucus bilayer.^28^ Depending on the experimental setup, shear stress generated by fluid flow might even direct cell fate along a specific path, as shown for the maturation/differentiation of human enteroids.^26^ Thanks to a relatively straightforward design and the fact that fluid flow can be applied based on previously described physiological parameters, the intestine-on-chip has since been widely adopted and used in different studies.^11^^,^^13^^,^^14^^,^^16^^,^^25^^,^^27^

Overall, microfluidics technology enables the development of complex cell culture systems with a variety of features that are hard to combine in traditional setups, improving physiological faithfulness. In fact, the translation of intestine-on-chip models to pharmaceutical studies could help decrease the burden of animal experimentation in the industry and, in the realm of personalized medicine, the development of patient-derived models for personalized drug testing from intestine-on-chips is a highly promising perspective. However, the translation from traditional cell culture to microfluidics-based platforms is not straightforward and requires extensive optimization of protocols owing to the use of distinct cell culture surfaces, reduced volumes of culture medium, different cell manipulation methods, and incompatibility with some common laboratory analysis equipment. In fact, scaling up and systematization of cell manipulation systems and assays for microfluidic cell culture platforms are future challenges associated with their practical application in the industry and the clinic.^45^ Key steps are being taken in this regard, with active partnerships already established between academia and several commercial companies to improve the manufacturing process and translating organ-on-chip devices from bench to bedside.^46^

## Mechanical Stimulation

The gut tube continuously is exposed to an array of mechanical forces early on during embryonic development and on a daily basis during post-natal life. In the digestive process, peristalsis is the involuntary and cyclic propulsion of food that occurs throughout the gastrointestinal tract generated by the smooth muscle in coordination with the enteric nervous system. Peristaltic movements aid in food digestion, nutrient absorption, and bowel emptying, but also induce shear stress and radial pressure on the epithelium.^47^ Importantly, these mechanical factors seem to influence intestinal development and homeostasis.

Intestinal looping, formation of villi, and the localization of crypts have been associated with mechanical strain.^48^, ^49^, ^50^ Moreover, chronic absence of mechanoluminal stimuli in the human intestine causes a decrease in ISCs as well as extensive alteration of epithelial microarchitecture,^51^ and is associated with pseudo-obstruction bowel syndromes. In a quite original approach, transplanted human intestinal organoids were exposed to mechanical strain in vivo by incorporation of a uniaxial spring inside their luminal space.^52^ Organoids derived from iPSCs and cultured in these conditions showed increased maturation as well as functional and transcriptional profiles similar to the native adult intestine. In the gut-on-chip,^10^ Caco-2 cells were exposed to peristalsis simulation provided by the cyclic elongation of a porous membrane. Although mechanical stimulation was not proven to be a determinant trigger for epithelial differentiation and the formation of villi-like structures, its association with fluid flow enhanced those responses. A similar technique for simulating peristalsis also was applied in a bioreactor model.^53^ Air was pumped into the external chamber, establishing controlled air pressure differentials between the internal and external chambers that caused the cyclic expansion and contraction of a highly stretchable membrane, exposing the tissue construct to mechanical stimuli. Using an intestinal channel with a unidirectional array of membrane valves, Cremer et al^54^ reported that peristaltic mixing also is necessary to prevent bacterial washout and for shaping the microbiota along the intestine. Mechanical stimulation also can affect the responses of the intestinal epithelium to other types of stimuli in vitro. For instance, upon exposure to heat-stable *Escherichia coli* enterotoxin, multidrug resistance protein 4 expression was up-regulated in human jejunal enteroids that were exposed to cyclic strain, resulting in increased cell secretion of cyclic guanosine monophosphate, a mediator of intestinal fluid homeostasis.^33^

Although the intestine is a mechanically active organ, not many model designs have explored this perspective, underestimating the impact mechanical stimulation has on cell organization and behavior. The limited number of studies available, which might be owing to a degree of undefinition concerning the interplay between dynamic mechanical forces and intestinal homeostasis, already highlight the importance of this parameter for proper intestinal in vitro modeling. The impact of different types of mechanical stimuli on intestinal culture should be assessed to evaluate if the current standard in intestine-on-chip systems—cyclic strain—is a faithful approximation to the native mechanical cues.

## Architectural Cues

The introduction of 3-dimensionality in cell culture was a crucial step for the construction of more relevant models of various tissues and organs. Two-dimensional (2D) cell culture is uncapable of fully recreating the physiological microanatomy of the tissue. As a result, the biochemical and mechanical cues present are different and influence cell behavior and communication. Generally, 3D cultures show greater cell viability, more physiologically relevant gene and protein expression profiles, as well as significant differences in proliferation, differentiation, and apoptosis processes, when compared with 2D cultures.^55^^,^^56^ Providing a 3D physical structure to cell culture can be achieved by embedding cells in a matrix that mimics the biochemical and mechanical characteristics of the native extracellular matrix (ECM) or by introducing microengineered surfaces. Therefore, results obtained on studies solely using 2D cultures may not be entirely reliable and can be avoided easily nowadays.

### Extracellular Matrices

In most reported 3D intestinal models, the frequent choice of ECM is a hydrogel, most notably Matrigel (Corning Life Sciences, New York, NY), a reconstituted basement membrane derived from extracts of Engelbreth–Holm–Swarm mouse sarcoma. Indeed, this commercially available bioactive matrix has been shown to effectively support intestinal epithelial growth and the formation of villi and crypt equivalents in gut-on-chip systems and in organoids.^6^ On the downside, clinical translation of Matrigel-based intestinal models is hindered significantly by several factors: xenogeneic origin, batch-to-batch variability, high cost, and poorly defined composition. Consequently, other natural matrices increasingly are being used to re-create the 3D microenvironment of the intestinal mucosa while overcoming these hurdles. For instance, alginate, an algae-derived polysaccharide, was shown to support the growth and development of intestinal organoids derived from human iPSCs, with an efficiency comparable with Matrigel, particularly at 1% and 2% (wt/vol) concentrations, with both types of organoids sharing a high degree of molecular similarity.^57^ Hence, alginate was proposed as a minimally instructive and cost-efficient alternative. However, this hydrogel was unable to promote growth and expansion of epithelium-only enteroids. On the other hand, Broguiere et al^58^ showed that a human-derived thrombin cross-linked fibrin gel provided appropriate support for the growth and development of intestinal organoids derived from isolated crypts, as well as single LGR5+ cells. In this setting, integrin binding to Arg-Gly-Asp adhesion domains (naturally occurring in the fibrin gel) together with the laminin-111/entactin complex (naturally present in Matrigel) were defined as the key mechanical and chemical determinants for organoid maintenance containing both stem and differentiated cells. This fibrin/laminin hydrogel also was shown to support long-term expansion of human liver and pancreas organoids, thus providing a simple 3D culture natural scaffold of defined properties and universal use.^58^ A collagen matrix was reported to induce intestinal organoid self-alignment and fusion, forming tubular multicellular structures.^59^ In addition, collagen has been used to culture intestinal epithelial cells on microengineered scaffolds with^17^ or without^60^ fluid flow.

Despite the success of some natural hydrogels in intestinal organoid culture, there is a recurrent search for synthetic alternatives. Synthetic matrices can be designed according to the specific characteristics and needs of the cells of interest. Different components can be incorporated in a hydrogel to improve mechanical stiffness, biochemical signaling, and matrix degradability. In addition, constructing in vitro tissue analogues using synthetic matrices is a modular process that grants mechanistic insight on how cells interact with the matrix and how each specific component or feature influences cell survival, proliferation, and differentiation.^61^ In this regard, the design of polyethylene glycol (PEG)-based hydrogels for intestinal organoid culture has been reported. Gjorevski et al^36^ based the conception of a mechanically dynamic matrix on the activity profile of yes-associated protein (YAP) as a readout along the process of differentiation and growth of the organoid. YAP is an effector protein of the Hippo signaling pathway that has been associated with cellular mechanosensing and ISC self-renewal. In stiff matrices, nuclear YAP activity decreased drastically over time, slowing organoid growth, whereas in Matrigel intermediate nuclear levels of YAP were constant over time. A higher stiffness appeared to be necessary at an initial stage for YAP activation and ISC expansion, but in vitro organogenesis seemed to require a progressively softer matrix, with reduced compressive forces. As a result, a hybrid hydrogel composed of a mechanically static PEG and a hydrolytically degradable PEG was produced to achieve a matrix that could soften over time and support organoid development.^36^ The dynamic nature of this hybrid PEG hydrogel simulates a typical process of cell–matrix interaction and continuous adaptation in 3D cellular microenvironments. In addition, this study also provided important clues on the interaction of intestinal organoids with the surrounding matrix, particularly at the molecular level, generating important information for further matrix engineering. Cruz-Acuña et al^62^ used a 4-arm PEG macromer with maleimide groups at each terminus for human intestinal organoid growth and expansion. In this hydrogel, adhesive Arg-Gly-Asp sequences were incorporated and collagen-derived GPQ-W (GCRDGPQGIWGQDRCG) protease degradable peptide sequences were used for cross-linking. A 4.0% hydrogel concentration was chosen because its mechanical properties were similar to Matrigel. Cruz-Acuña et al^62^ proved that the decrease in organoid viability at higher polymer densities was the result of a change in mechanical stiffness of the matrix and not the result of the change in permeability of the hydrogel, showing that the window of mechanical properties that supports intestinal organoid growth is quite narrow. This hydrogel successfully supported the growth and differentiation of human iPSC-derived intestinal organoids and was used as an injection vehicle of human intestinal organoids that improved repair of intestinal and colonic wounds.

### Microfabricated Scaffolds

Microfabrication techniques are progressively being translated from the field of electronics to tissue culture with the goal of introducing novel features in tissue engineering approaches that improve the recapitulation of the original microenvironment of the tissue. When constructing scaffolds for cell culture, microfabrication techniques can be used for creating specific 3D structures as well as topographical or molecular patterns on cell culture surfaces.

In the case of intestine-on-chip models, the villi of the intestinal mucosa has been re-created mostly by micromolding.^17^^,^^30^ Although these studies have shown that the combination of fluidic flow with the re-creation of the intestinal 3D microarchitecture induced Caco-2 cells to behave more similarly to the native tissue, specifically in terms of enzymatic activity, distribution of proliferative and apoptotic cells, and glucose transport, the crypt compartment was not re-created. Wang et al^60^ created a micromolded collagen scaffold that mimics the topology (ie, villi and crypts) of the intestinal mucosa. Biopsy-derived intestinal cells organized themselves in a monolayer on the cross-linked collagen surface in static condition and the design enabled the compartmentalization of a stem cell zone and a differentiated cell population, with proven unidirectional migration of cells to the villi. Nikolaev et al^20^ developed an organoid-derived tubular intestinal model using a microfabricated scaffold coated with a hydrogel mixture of collagen and Matrigel. Laser ablation was used to create the microchannels that mimicked the intestinal lumen and crypts. ISCs self-organized into a functional epithelium with a lumen and the system was maintained under repeated intraluminal perfusion. The intestinal tube-shaped structures showed an extended lifespan in comparison with cystic organoids, the presence of rare epithelial cell types not commonly found in other 3D models, and self-regenerative capacity. The intestinal biochemical environment has been recapitulated in models of human small intestine^60^ and colon^63^ using microengineered collagen scaffolds. These platforms enable spatial and temporal control of signaling gradients and may provide new information about intestinal morphogenesis, stem cell maintenance, crypt dynamics, and epithelial regeneration. Moreover, exposure to gradients of microbial products and inflammatory cytokines is relevant to understand the impact of these factors on tissue homeostasis. Ultimately, growth factor gradients can aid in enhancing the mimicry properties of in vitro intestinal models such as the case of intestinal organoids that form irregular structures consisting of an enclosed lumen with buds, lacking a physiologically relevant shape.

Recently, rapid prototyping techniques have been introduced in this field owing to their low cost, simplicity, design flexibility, and improved resolution. One of those is 3D printing, a group of manufacturing methods based on layer-by-layer material deposition with the final goal of creating a 3D object.^64^ A stereolithography-compatible hydrogel, composed of PEG-diacrylate, acrylic acid, and fibronectin, was developed to create support with intestinal topography,^65^ comprising villi-like protrusions and crypt-like invaginations. In comparison with 2D culture on the same hydrogel, differentiation and polarization of Caco-2 monolayers were enhanced in the villi domains of the 3D scaffold, although crypt compartments were not developed successfully in either condition.

In summary, Matrigel is used extensively in microfluidic models of the gut, which is associated with its bioactivity and showed potential for supporting intestinal epithelial culture. However, the use of Matrigel hinders the potential clinical and pharmacologic translation of gut-on-chip models owing to animal origin and variability and, for that reason, it is expected to be progressively replaced in the coming years by composition-defined matrices. Alginate, laminin, collagen, and PEG-based hydrogels are compatible with intestinal epithelial growth and differentiation and should be adopted in future intestinal models. PEG-based synthetic hydrogels are particularly interesting because their chemical, biological, and mechanical characteristics are more tunable. The combination of optimized matrices with microengineered scaffolds in intestinal culture, as previously attempted by some groups,^20^^,^^65^ is a more advanced approach by simultaneously re-creating the 3D microarchitecture of the tissue and the biochemical activity of the ECM, possibly inducing a synergistic effect on the physiological similarity of intestinal models.

## Co-culture Systems

A healthy intestine is maintained by a complex interplay between different tissues and cell populations. In fact, the absorption of nutrients in the intestine is only possible thanks to: the close contact with endothelial cells and blood circulation; the presence of microbiota in the mucosa that enhances epithelial barrier resistance, nutrition and immunity; the constant invasion by immune cells that protects the mucosa from pathogens; the proximity with mesenchymal cells that is important for the stem cell niche and to provide physical support to the epithelium; and the muscle and nerve cells that control peristaltic movements and satiation. It has become evident that in vitro models of the human intestine must evolve to recapitulate the different actors involved in intestinal homeostasis, infection, and pathology.

### Microbiota

The group of all microorganisms that populate the intestinal mucosa—the microbiota—establish a symbiotic relationship with the intestinal mucosa and benefit human health. They produce enzymes that are not produced endogenously, which is helpful in digesting complex nutrients, they influence xenobiotic metabolism and interfere with drug pharmacokinetics, either reducing or enhancing their effect.^66^ In addition, low bacterial diversity has been associated with inflammatory bowel disease (IBD), allergies, obesity, and diabetes, given that the presence of commensals induces the epithelium to produce antimicrobial components, and, overall, creates a balance between toleration of microorganisms and prevention of pathogenic growth.^67^ The intestinal host-microbiota interaction already has been studied in 2D cultures and in Transwell devices,^68^^,^^69^ but bacterial overgrowth and consequent epithelial cell death limit the duration of these experiments to a few hours. Thus, co-cultures in advanced 3D models are being explored to mimic intestinal microbiota and host interactions.

Co-culture of intestinal epithelial cells and microbiota in a microfluidic chip was reported by Kim et al.^10^ The co-culture of Caco-2 cells with *Lactobacillus rhamnosus* GG under cyclic mechanical strain and fluid flow was successful, with both populations remaining viable after 96 hours. This gut-on-chip circumvented the bacterial overgrowth issue by providing continuous perfusion, which eliminates nonattached bacteria and dilutes acidic metabolites. Interestingly, this study underscored the importance of the microbiota in intestinal homeostasis because transepithelial electrical resistance measured in the gut-on-chip significantly increased over time, which translates to an improvement of intestinal barrier integrity. Such an increase shows that the microbiota releases biochemical cues that stimulate epithelial cells and enhances their functions, contributing to a healthier intestine. Shah et al^12^ proved that long-term co-culture of Caco-2 cells with aerobic and anaerobic commensals induces transcriptional and metabolic alterations in intestinal cells, related to the species present. Although most studies focused on the host-microbiota interaction include only 1 type or a defined group of microorganisms, a recent study focused on the co-culture of intestinal epithelial cells with complex microbiota samples extracted from feces, including aerobic and anaerobic species.^11^ After 3 days of co-culture, bacterial diversity in the chip was similar to bacterial diversity found in intestinal aspirates.

Intestine-on-chip models also may be interesting platforms to study the mechanisms of pathogenicity of diverse microorganisms. An in vitro model of intestinal *Shigella flexneri* infection was developed successfully by Grassart et al.^31^ This study showed that the bacteria accumulate preferentially in crypt-like domains, similarly to what happens in vivo, and that invasion efficiency and propagation are highly enhanced by the mechanical forces of the intestinal microenvironment, cyclic strain, and shear stress. Bacterial invasion occurred in a directional fashion and caused loss of villi-like structures and epithelial destruction. These results suggest that the 3D structure of the intestinal epithelium and its dynamic mechanical environment contribute to the efficiency of *S flexneri* infection, justifying the failure of a Transwell-based model. In addition, another study constructed an interface model of colon epithelium and endothelium to investigate the higher susceptibility to enterohemorrhagic *E coli* in human beings in comparison with mice. It was possible to correlate this observation with the enrichment of 4 specific human microbiome metabolites that cause an induction of chemotaxis and motility-related genes, increasing bacterial infectious activity.^32^

### Immune Cells

Other studies have investigated the immune component in intestinal physiology, mostly in association with the microbiota during inflammatory settings. Because of the nature of inflammatory processes in the intestine, the role of other cell types cannot be disregarded, thus, some of those models include peripheral blood mononuclear cells (PBMCs) and endothelial cells. Kim et al^13^ established an IBD model using the aforementioned gut-on-chip system by co-culturing pathogenic or nonpathogenic strains of *E coli* and PBMCs. The presence of PBMCs exacerbated the impact of bacteria in the epithelium, causing destruction and shortening of the villi and hindered barrier integrity. A similar model suggested that patients with a leaky gut are significantly more vulnerable to microbial infections and aggressive immune activity owing to higher permeability of the epithelial barrier, which can turn into chronic inflammatory conditions.^14^ On the other hand, the MOTiF (Multi-Organ-Tissue-Flow; microfluidic ChipShop GmbH, Jena, Germany) device is made of a cyclin olefin copolymer and consists of an upper and a lower channel, separated by a porous polyethylene terephthalate membrane.^15^ To re-create the role of immune cells in the intestinal mucosa, primary monocytes that later differentiated into mucosal macrophage and dendritic cells were seeded on top of the confluent endothelial layer in the upper channel, while Caco-2 cells were cultured in the lower channel. Co-culture with *L rhamnosus* induced immunotolerance, protecting the epithelium from the inflammatory effects of lipopolysaccharide exposure and opportunistic infections. Moreover, the OrganoPlate model^22^ (Mimetas, Leiden, the Netherlands) also has been applied to the replication of the interaction between intestinal epithelium and immune cells.^35^ By combining primary-derived epithelium with matrix-embedded macrophages, Beaurivage et al^37^ built an immunocompetent intestinal mimic. In response to a lipopolysaccharide and interferon γ trigger, macrophages polarized into the M1 proinflammatory phenotype, whereas epithelial cells responded with the release of epithelial inflammatory cytokines. In a similar approach, neutrophils also were incorporated in the circulatory compartment of the OrganoPlate model. Neutrophils were capable of infiltrating the hydrogel channel in inflammatory settings, a response that was exacerbated in the presence of macrophages and caused epithelial damage.^70^

### Endothelial Cells

Several models of intestine-on-chip also have recapitulated the epithelial–endothelial interface by culturing an endothelial monolayer on the opposite side of the membrane where epithelial cells are seeded.^11^^,^^15^^,^^16^^,^^25^ In this configuration, when both compartments are perfused simultaneously, the systemic circulation and luminal shear stress is re-created. Kasendra et al^25^ reported that the co-culture of duodenum-derived organoid fragments with human intestinal microvascular endothelial cells accelerated the formation of the epithelial monolayer from 6 to 2 days, and increased the efficiency of monolayer formation from single cells. On the other hand, the co-culture with endothelial cells did not significantly affect the permeability of the intestinal barrier.^11^^,^^25^ The simulation of the epithelial–endothelial interface is particularly important when studying nutrient absorption and for pharmacologic assays. Recently, the previously mentioned model was assessed as a platform for the study of drug transport, metabolism, and drug–drug interactions in human beings.^16^ Because the focus of these studies was not the interaction between the epithelial and endothelial compartments, it is impossible to assess whether the results obtained, such as the closer transcriptomic similarity of these models to the human intestine in vivo, is a direct consequence of the endothelial co-culture. In future microfluidic-based models of the intestine, characterizing this type of co-culture concerning its impact on the morphology, functionality, and protein expression of the intestinal epithelium would be important to dissect and explain the results obtained in previous works.

### Mesenchymal Cells

Intestinal mesenchymal cells, including fibroblasts, myofibroblasts, and smooth muscle cells that reside in the lamina propria, have been associated with crucial intestinal mucosal activities.^71^ Mesenchymal cells secrete morphogenetic factors and contribute to the stem cell niche.^72^, ^73^, ^74^ The Wnt pathway is the critical signaling hub responsible for stemness maintenance in the bottom of the crypt and mesenchymal cells are involved in its regulation by producing Wnt ligands, R-spondins, Wnt antagonists, and BMPs. Platelet-derived growth factor receptor α, Forkhead box L1, and GLI family zinc finger 1 proteins are proposed markers for the identification of mesenchymal cell populations that contribute to the ISC niche.^72^, ^73^, ^74^ Although the relevance of nonepithelial populations for the stem cell niche now is becoming recognized, it appears to result from a collective effect of cells with distinct phenotypes and functional redundancy. Mesenchymal cells therefore are essential for establishing signaling gradients, where the base of the crypt has active Wnt signaling and epithelial differentiation along the villus is partially regulated by other pathways, including BMP signaling. These cells also are involved in the immune response of the mucosa, being considered nonprofessional immune cells.^71^ In an inflammatory scenario, intestinal myofibroblasts and fibroblasts secrete soluble cytokines, chemokines, and growth factors to recruit professional immune cells to the site of infection, modulating the type and magnitude of the immunologic response. In fact, the mesenchymal compartment also is involved in some gastrointestinal diseases (ulcerative colitis, celiac disease) via alteration of matrix deposition, up-regulation of proinflammatory cytokine production, and imbalance of mucosal inflammatory cells.^71^

Co-culture experiments of intestinal epithelial and mesenchymal cells have been reported in the literature, providing insights on the mechanism of interaction between the 2 compartments. 2D co-culture with fibroblasts or myofibroblasts promotes epithelial proliferation and differentiation by paracrine action of keratinocyte and hepatocyte growth factors.^75^^,^^76^ In an air–liquid interface culture system, co-culture of myofibroblasts was shown to extend the viability of murine neonatal intestinal epithelium and promote multilineage differentiation, without external addition of growth factors associated with the ISC niche.^77^ An efficient process to differentiate human iPSCs into intestinal tissue has been established, resulting in the formation of 3D organoids with crypt–villus-like structures and a surrounding mesenchyme.^42^ This approach is a step forward in the enhancement of the physiological relevance of organoid models of the intestine, by incorporating an additional cellular type that is involved in intestinal homeostasis. Although the importance of the mesenchyme to intestinal physiology has been recognized before, this type of co-culture has not been explored significantly in gut-on-chip models. In 2020, a novel study showed that intestinal subepithelial myofibroblasts had a potent pro-angiogenic effect in the intestine and enabled the organization of endothelial cells into hollow vessel-like structures.^38^ The co-culture of the perfused capillary structures with intestinal epithelial cells in a gut-on-chip device resulted in the re-creation of systemic circulation and a luminal compartment where a polarized epithelium was formed.

### Nerve Cells

The complex neuronal network associated with the gastrointestinal tract is named the enteric nervous system (ENS). The ENS controls various intestinal activities, such as the patterns of movement of the gastrointestinal tract, gastric acid secretion, movement of fluid across the epithelium lining, changes in local blood flow, nutrient handling, and interacts with the immune and endocrine systems of the gut.^78^ The ENS has bidirectional connections with the central nervous system, and works in concert with it to control the digestive system in the context of local and whole-body physiological demands.^79^ The ENS is composed of ganglia, nerve fibers that connect ganglia, nerve fibers that supply the muscle of the gut wall, the mucosal epithelium, arterioles, and other effector tissues.^79^ In the small intestine, enteric glia reside around crypts and throughout intestinal villi and neurons are located under the crypts in the submucosa and between smooth muscle layers.^80^ The ENS is thought to be linked with the intestinal epithelium by neuronal and glial projections that connect to enteroendocrine cells.^81^

Studies have focused on the interplay of the intestinal mucosa with the ENS. Enteric glial cells release transforming growth factor β1, which inhibits epithelial cell proliferation, being important regulators of intestinal barrier function.^82^ Furthermore, enteric glial cell ablation leads to disruption of the intestinal barrier. A Transwell platform was adapted to the co-culture of intestinal cells with enteric neurons, glia, and myofibroblasts.^83^ Results showed that this co-culture promoted an epithelial enrichment of the enteroendocrine lineage, evident by an increase in the number of cells expressing chromogranin A. In addition, the production of cytokines by the ENS compartment was increased significantly, in comparison with the ENS monoculture. These findings suggest the ENS partially modulates epithelial proliferation, differentiation, barrier integrity, and inflammatory response. Alterations in this crosstalk might cause intestinal pathologies and can be exploited for therapeutic interest.

Westphalen et al^84^ postulated that tuft cells, a rare type of intestinal epithelial cells, are mediators of neural signals to the intestinal mucosa and can survive only in close proximity to neurons. Tuft cells are absent in intestinal organoids, and they are not necessary for their growth and maturation. However, regeneration upon tissue injury is hindered significantly in the absence of tuft cells. Another study developed a protocol for the construction of iPSC-derived intestinal organoids associated with nervous tissue derived from neural crest cells.^85^ Transplantation of the 3D construct in vivo allowed the migration of the neural crest cells to the mesenchyme where they self-organized and differentiated into multiple cell types of the ENS. The nervous network formed comprised ganglionic structures and interganglionic fibers that resembled the embryonic development of the myenteric and submucosal neural plexuses. The ENS mimic also proved to be functional by the formation of a wave of contractions, independent from muscular contractions, when exposed to low voltage. By introducing mutations on the paired-like homeobox 2B transcription factor, researchers created a model of Hirschsprung’s disease, which causes aganglionosis in the intestine. Hirschsprung’s disease–like intestinal organoids transplanted in mice showed impaired smooth muscle and nervous development and a lower engraftment success rate than wild-type–derived organoids.

Constructing a microfluidic model with all of the different cell types that interact in intestinal physiology might be utopic, owing to the high level of complexity. In intestine-on-chip systems, the co-culture with microorganisms, and endothelial, immune, and mesenchymal cells already has been achieved. Further steps in co-culture systems could focus on investigating the interaction of smooth muscle and nerve cells with the intestinal microenvironment given that these populations are actively involved in various intestinal activities such as motility and communication with the immune and endocrine systems. Globally, the crosstalk between the epithelial and mesenchymal compartments also has been neglected in in vitro intestinal models. A deeper focus on this subject could help to clarify uncertainties regarding the contribution of individual types of mesenchymal cells to the ISC niche. Simple culture systems first should be studied and optimized and, gradually, different cellular components should be added to create progressively more complete in vitro models that accurately can mimic the contributions of the various cell types to intestinal homeostasis and disease.

## Oxygen Gradient

The intestinal epithelium is heavily supplied with oxygenated blood. The proximity with the circulatory system is crucial to enhance nutrient absorption in the villi and to expedite immunologic responses against pathogens. Because of the countercurrent blood flow in the villi and the microbial metabolic activity in the mucosa, an oxygen gradient is established across the intestinal wall. Oxygen concentration decreases in a radial axis from the intestinal submucosa to the lumen, where the microbiota reside.^86^ Despite the discrepancy of different measures in the literature, estimates show that oxygen tension decreases from 59 mm Hg in the base of the crypt to 22 mm Hg at the villus tip and to less than 10 mm Hg in the small intestinal lumen.^87^ This value changes along the intestine, with the sigmoid colon lumen reaching extremely low values such as 3 mm Hg.^88^ The hypoxic environment of the intestinal lumen appears to be generated because of the breakdown of dietary nutrients together with oxygen consumption by oxygen-tolerant microorganisms, enabling anaerobes to proliferate in the intestinal lumen. Indeed, variations in luminal oxygen concentration alter the composition of the gut microbiota and can lead to dysbiosis.^89^ Hypoxia induces production of hypoxia-inducible factors (HIFs) by luminal adjacent epithelial cells. In turn, HIF stabilization regulates the expression of genes involved in barrier integrity, energy metabolism, and angiogenesis, being responsible for overall cellular adaptation under these conditions.^86^ On the other hand, an overactivation of the hypoxic/HIF gradient is associated with pathologies such as IBD and cancer.

The intestinal anoxic–oxic interface has been difficult to re-create in vitro owing to the use of conventional aerobic cultures. Although the epithelium contributes significantly to the establishment of an oxygen gradient, it is by itself insufficient to support anaerobe growth. Intestinal models in this context have been established mainly to allow the co-culture of obligatory anaerobes in host-microbiota infection studies. In most of these models, the gradient forms across 2 separate compartments, which represent the intestinal lumen and the capillary vasculature, and the 2 typically communicate through a gas-permeable membrane. A Transwell-based static design such as this one was first used to co-culture the obligate anaerobic strain Faecalibacterium prausnitzii with Caco-2 cells, but supported only a short-term (12 h) co-culture period.^90^ Another co-culturing system (HoxBan model) used solid agar medium for bacterial growth overlaid with culture medium exposed to air for Caco-2 cells, but also was analyzed after 18–36 hours.^68^ Afterward, in the intestine-on-chip model HuMiX (human-microbial crosstalk), it was observed that the establishment of a hypoxia gradient by perfusing oxygenated medium through the subepithelial channel, while anoxic medium circulates in the luminal compartment, allows co-cultures between Caco-2 cells and anaerobic bacteria, recapitulating in vivo transcriptional, metabolic, and immunologic signatures.^12^ The establishment of a controlled oxygen gradient in intestine-on-chip systems recently was shown to support direct co-culture of intestinal epithelial cells with complex communities of anaerobic and aerobic human commensal gut bacteria^11^ and their sustained viability in long-term culture.^34^^,^^29^

Thus, the oxygen gradient established in the intestine is clearly involved in several physiological functions and pathologies and also should be incorporated in intestinal in vitro models. Microfluidic platforms are crucial tools to improve the simulation of this interface in the long term, together with the simultaneous co-culture of aerobic and anaerobic microorganisms.

## Conclusions and Future Perspectives

Intestine-on-chip models have the potential to combine all the characteristic features of the native intestine into one miniaturized, self-contained, and portable culture system. Some of the major breakthroughs attained with intestine-on-chip models thus far have been the self-organization of cells into the native microarchitecture of the intestine, the acceleration of epithelial differentiation, long-term co-culture with microbiota, and the intraluminal perfusion of organoids. Nevertheless, there still are significant hurdles to overcome (Table 2).

**Table 2:** Current Outstanding Questions and Future Challenges for Intestine-on-Chip Models

Parameter	Outstanding questions	Future challenges
Fluid flow	How to re-create tissue level–morphogen gradients?	Achieve simultaneous distribution of compartment-specific media formulations (universal culture medium?)
Architectural cues	What is the best material to instruct/sustain intestinal stem cells homeostatic balance?	Define an all-in-one intestinal 3D scaffold (crypt–villus axis)
Mechanical stimulation	What is the specific contribution of mechanical cues to intestinal identity and differentiation?	Emulate peristalsis-like motion
Co-culture	How many cell types should be included to achieve (minimal) functional tissue organization?	Optimize culture conditions for long-term maintenance of ≥3 different cell types
Oxygen gradient	How to quantify and regulate oxygen tension in a microphysiological environment?	Create sustained oxygen gradients for aerobic/anaerobic interface cultures using human primary intestinal cells

The choice of study model is a critical point, with distinct cellular sources available that need to be considered carefully in light of experimental goals. For instance, research on intestinal barrier function and drug absorption already can benefit from the use of human primary intestinal cells to progressively replace the generalized use of immortalized cancer lines, notably Caco-2 cells. Human intestinal organoids (or their malignant counterparts) seem the most practical approach for applications involving the interrogation of mature tissue. However, the scarce amount of human biological material directly obtained in a clinical setting (ie, biopsies) is a limiting step. This should be bested with the regulated dissemination of biorepositories of high-quality standards, allowing expansion of primary cells as enteroids, colonoids, and intestinal tumoroids.^91^ On the other hand, iPSCs-derived intestinal tissues have an indisputable value for developmental biology and disease modeling, with ease of availability and the possibility of generating nonepithelial cell types. But guiding iPSCs through a not yet fully understood regimen of germ layer– and lineage-specifying culture conditions might pose risks when aiming for a future regenerative intent, namely the presence of off-target cells or immature fetal-like tissue. A shift toward the use of intestine-on-chip models using organoid-derived intestinal cells seems evident over the past 2 years, highlighting the benefits of combining both systems.^16^^,^^25^ Because major intestinal pathologies such as IBD (6–8 million new cases worldwide) and colorectal cancer (third cause of cancer-related deaths globally) mainly affect the colon, this justifies investing in the specific development of an advanced system supporting the long-term maintenance of human colonic tissue to be used as a physiologically relevant preclinical model.

A topic of intense research for the coming years will certainly be the optimization of microfabricated platforms integrating different cell populations at once while maintaining ease of use and the ability to interpret results. Particularly challenging will be the introduction of the endothelial and immune components in these systems: the former will allow re-creating a functional vasculature and overcoming the diffusion limit to attain larger tissue mimics, key for implantation purposes; the latter will allow the establishment of immunocompetent tissue constructs, recapitulating both healthy gut physiology or diseased inflammatory states. Importantly, reproducibility and success of the different co-culture systems is highly dependent on the architectural, mechanical, and biochemical features of the model. Also, the type of material used in the device will have an impact.^92^ For instance, it is widely recognized that there is an issue of nonspecific binding of small hydrophobic molecules onto polydimethylsiloxane surfaces,^93^ the most commonly used polymer for intestine-on-chip fabrication to date. Hence, a modular approach should be taken to avoid future setbacks, in which it is essential to attain knowledgeable control over one component before introducing the next one. Significant advances already made in defining tissue-like topography,^60^^,^^63^^,^^65^ non–animal-derived ECM substrates sustaining ISC self-renewal and differentiation responses,^36^^,^^62^ and morphogen gradients mediated by fluid flow^27^ might allow the establishment of standard operating procedures for intestine-on-chip epithelial cell culture in the short term. Sustained progress toward increasingly more complex models might depend on different research groups using their complementary expertise to study the interaction with additional cell types while doing it from a defined common starting point.

In conclusion, although significant strides have been made over the past years concerning microfabrication, microfluidics, and primary cell culture techniques for the design of intricate intestine-on-chip devices, additional steps still are required to achieve the ultimate ex vivo model of the human gut. Until then, the models already established and the ones to be developed hold great potential, not only helping to reduce the toll on animal testing but also advancing basic research, laying the groundwork for physiologically more relevant preclinical studies and the advent of personalized medicine in gastrointestinal diseases.
